# Flower reflectance and floral traits data from Ökologisch-Botanischer Garten (OBG), Germany

**DOI:** 10.1016/j.dib.2024.110512

**Published:** 2024-05-11

**Authors:** Mani Shrestha, Evelyn Hlawatsch, Hannah Pepe, Louis-Marvin Sander, Dietmar Schreier, Max Schuchardt, Andreas von Heßberg, Anke Jentsch

**Affiliations:** a Department of Disturbance Ecology, Bayreuth Center of Ecology and Environmental Research (BayCEER), University of Bayreuth, 95447 Bayreuth, Germany; b Faculty of Biology, Chemistry and Earth Sciences, University of Bayreuth, Universitätsstr. 30, 95447 Bayreuth, Germany; c Department of Life Science, National Taiwan University, Taipei, Taiwan

**Keywords:** Angiosperm, Flower colour, Plants, Pollinators, Reflectance, Vision

## Abstract

Not all colours are perceived and interpreted equally. The electromagnetic spectrum is perceived differently by the distinct visual systems of animal species, resulting in differences in each species’ colour perception. Given the diverse colours found in flowering plants, it is interesting to consider the colour perception of insects and the co-evolution of flowering plants to attract pollinators. Here, we considered the differences between human visual systems and that of bees and flies—the two largest insect pollinator groups. We collected flower reflectance spectral data of 73 species across seven human-perceived colours using a spectrophotometer. Minimum of 3 different flowers were used to measure the reflectance properties of flower colours. The raw data can be used to visualize the different animals’ visual systems i.e. it can be processed and translated into known photoreceptors of human, bee, and fly visual systems. Overall, our data will help to compare how different animals see flower colours in the natural world and will also highlight the importance of understanding the interspecific communication in plant-pollinator communities. Thus, our data will assist scientists in the future to recognize the floral colour evolution in angiosperms.

Specifications Table

**Table:** 

Subject	Biodiversity, Plant-pollinator interactions, Ecology
Specific subject area	Visual ecology of insects and plants signal
Type of data	RAW/ Processed
Data collection	Data collected randomly based on human visual colour vision to test the how different animal see the colours. Sample were brought to the lab for the spectral measurements. We acquired the data using *Ocean Optics Spectrophotometer* with *OceanView* software version 2.0.8 attached to the PC. We collected the flower sample on its peak flowering season June to August.
Data source location	*Region*: Bayreuth, Germany
	*Location*: Ökologisch-Botanischer Garten (ÖBG)
	*Coordinates of sampling sites*: 49.9257°N, 11.5842°E
Data accessibility	All of the R scripts used for processing the data are available in public domain.
	*Repository name*: **figshare**
	DOI:10.6084/m9.figshare.21081025.v2
.	*Instructions for accessing these data*: Data is already available without any restriction in figshare

## 1 Values of the Data

• Data will help to identify the different ways in which flower colours are viewed between humans, bees, and flies or other animals.
• Flower colour is perceived differently by the human visual system than by that of insect pollinators (e.g., bees and flies) due to differences in photoreceptors sensitivity and the inability of humans to perceive UV light compared to bees and flies. Thus, data can be used to understand the how different animals see the world.
• Data can be used in global research on floral colour and pollinator vision, biodiversity conservation. Getting a spectral data for the flowering plants in remote location, it is almost impossible either due to equipment unavailability or lack of trained scientist. As a result, the information gathered from flowering plants growing in botanical gardens is always helpful while conducting global or macro-ecological meta-analysis/ or original research. In our instance, we want to incorporate these data's in flower colour evolution across the angiosperms data analysis and prepare the global floral colour database.

## 2 Background

It is often easy to forget that other species do not always perceive the world as we do and may see a different reality than us humans. This is especially true when we consider the colour spectrum that we perceive as visible light, which other animals, such as insects, experience differently. While many flowers are often visually appealing to us, they may also be visually alluring to insects such as bees and flies, but in a different way. By studying the vision of major pollinators (i.e., bees and flies), we as humans can compare the way in which flower spectral data may be viewed by these insects and by ourselves.

There is much diversity among flower traits and colours, with flower colour being a symbol of diversity within angiosperms and often a means of plant species identification [1]. But flower colour serves a greater purpose than just looking appealing to humans. Flowering species have evolved with different colour signals in order to attract different pollinators [2] and to increase detection rates [3] by the visual systems of these important pollinators [1,4]. Indeed, between 100,000 and 200,000 different animal species are involved in pollinating the ∼250,000 species of wild flowering plants, including insects, mammals, and birds [5]. However, there are differences even between insect groups in visual detection of flower colours(e.g. flies being tetrachromatic and bees are trichromatic [[4], [6], [7], [8]] Humans also have a trichromatic visual system, with photoreceptors maximally absorbing wavelengths of red, green and blue light, but we still differentiate from bee visual systems in that bees have photoreceptors that are maximally sensitive to green, blue and ultraviolet (UV) light [[9], [10], [11]].

Flower spectral data collected form the botanical garden are already processed (data ranged from 300 to 700 nm) to make easier to use. Data also useful to use for visual colour modelling of tri- and tetra-chromatic colour vision models for humans, bees (e.g. *Apis mellifera* and *Bombus* sp), and flies (*Eristalis t*enax) as they are more commonly used in agroecological settings besides natural pollinations. Other details are provided below at ‘Experimental design, materials, and methods’ sections.

## 3 Data Description

**Fig. 1:**
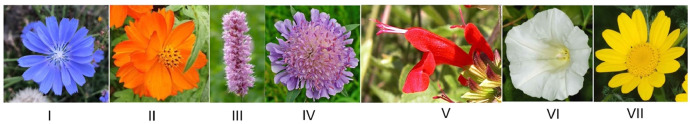
Examples of the 7 different colour flowering species. From left to right: I) blue: *Cichorium intybus*, II) orange: *Cosmos sulphureus*, III) pink: *Bistorta officinalis*, IV) purple: *Knautia arvensis*, V) red: *Salvia coccinea*, VI) white: *Calystegia sepium* and VII) yellow: *Costa tinctorium*.

**Fig. 2:**
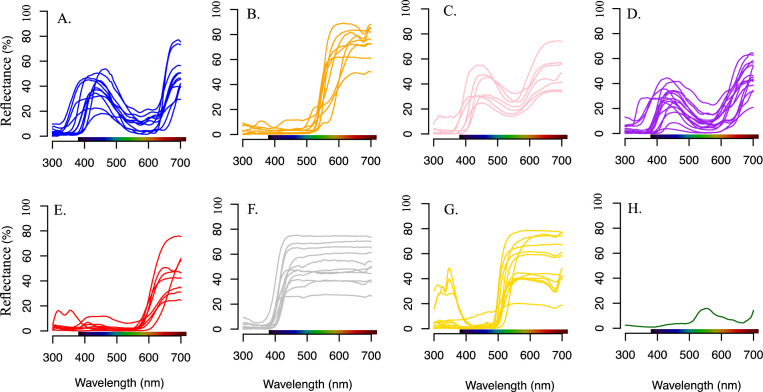
Reflectance spectra of sampled flower colour groups of different flowering plant species. A. blue flower; B. orange flower; C. pink flower; D. purple flower; E. red flower; F. white flower; G. yellow flower; and H. average green leaves. Colour bar in x-axis represents human vision ranges.

We provide all the data in the form of data table. All data's are available on *Figshare* [12]. Data on figshare were labelled “data_&_rcode_Shrestha_et_al .zip”. Within this folder, data for green leaves are on ‘lvs’ folder, data for flowers of different colour groups (Fig. 1) are on folder ‘petals’. Leaves folder ‘lvs’ contains average green leaves spectral data (first column with wavelength 300–700 nm and second column with average leaves spectral data on percentage,%). Each ‘.csv’ file in the folder ‘petals’ are labelled based on human colour categories. Each file contains Multiple species and each column except first column are labelled with species name. Spectral data contains wavelength 300–700 nm range (first column) and rest of the other column contains spectral reflectance's (%) data for each flower species. R code also available on main folder with read me file about the data structure. The data was processed as described in the methods section. Processed spectral reflectance provided in Fig. 1 and process of marker point calculation is provided in Fig. 2.

## 4 Experimental Design, Materials and Methods

### 4.1 Data collection

We collected flower samples of different plant species from the botanical garden in Bayreuth, Germany (Ökologisch-Botanischer Garten, 49.9257°N, 11.5842°E). The flower samples (*n* = 73) were collected across seven different human-perceived flower colours —blue, pink, purple, orange, red, white, and yellow (Fig. 1) — over the summer of 2021 (June to September).

At least three to five freshly opened, intact flowers were selected for all the colour categories, and when possible, correctly identified specimens in the botanical garden were collected and further identified using the *Flora Incognita* [13] and *Pl@ntNet* [14] identification apps. All identifications were cross-checked using local flora guides [15]. The flower samples were transported immediately to the lab in plastic zipper bags for flower colour reflectance spectra measurement. We collected blue (*n* = 11), orange (10), pink (10), purple (17), red (10), white (11) and yellow (14) plant species (examples: Fig. 1).

### 4.2 Flower colour measurement

We measured the flower reflectance spectral data from 300 to 700 nm wavelength using the Ocean Optics spectrophotometer (USB2000+UV–vis-ES, Ocean Optics, Dunedin, FL, USA), a Xenon light source, and Ocean View Software connected to a PC. The flower petals were fixed onto a black background during measurement so that the background did not influence the measurement. The sensor was placed 5–7 mm away from the petal's surface using a sensor holder, and the measurements took place at a 45° angle. At least three flowers were measured relative to a Lambertian, PTF WS-1 reflectance standard (Ocean Optics) following the method used in [2] to generate multiple reflectance profiles for each species. We calibrated our equipment before each flower measurement to avoid the drift on calibration due to external lights and to reduce electric noises. Large flowers were measured at the top, middle, and bottom of the petal. For small flowers, several petals were fixed adjacent to each other and then measured. For the Asteraceae family, only the ray florets were measured.

An average leaf-reflectance of multiple species (human green) was used as the background for the colour modelling. Exact setup and handling instructions can be found in the official manual of the spectrometer device [17]. Sampled flowers and leaves spectrum under human colour groups are given in Fig. 2.

### 4.3 Data processing and smoothing of spectral data

**Fig. 3:**
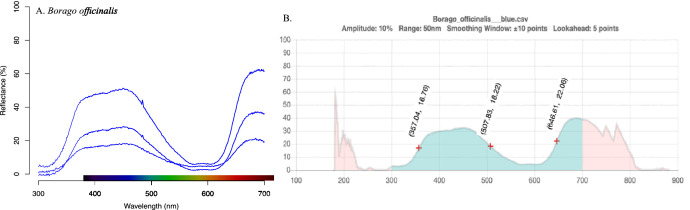
Example of reflectance spectral data and marker point calculation: A) unsmoothed raw reflectance spectra for the petals of Borago officinalis, colour bar on x-axis represents human vision. B) smoothed spectrum and process of marker point calculation, plus (+) symbols are the marker points where bees best discriminate the flowers colour.

The next step is referred to as “smoothing”. In this process, our data was fed into a program that post-processed our data output by removing the electric noise produced while measuring each species’ flower reflectance spectra. We used a method developed by [18,19] for data smoothing and evaluation. The smoothing process uses local regression of the mean value of the three replicates, each at the same wavelength, to generate a single result curve (“uniformly weighted averaging”, see Fig. 3A and B).

### 4.4 Marker point calculation and used parameters

We used the smoothed spectral data to find the important marker points using a method implemented by [19]. In this calculation, the spectral curve was cut into monotonic increasing and decreasing sections. In these areas, the algorithm searched for points/wavelength sections where the in-/decrease in reflectance was more than 10 % reflectance at 50 nm intervals for a specific minimal wavelength section (details available in [2,16]). The points identified by the algorithm were located in the middle of the monotonic in-/decreasing areas (see Fig. 3B).

### 4.5 Colour visual modelling (Data transformation for visual model)

For the visual colour modelling, we used the smoothed reflectance spectra (Fig. 2).; Our collected data ranged from 300 to 700 nm. As the photoreceptors of honeybees and flies do not capture values above 650 nm, we only used adapted reflectance data between 300 and 650 nm wavelength in our colour visual modelling.

We plotted all the flower colour data in tri- and tetra-chromatic colour vision models for humans, bees, and flies. The calculations for the human, bee (*Apis mellifera*) and fly (*Musca domestica)* colour models were performed using the package “PAVO” [[20], [21]]. For all three models, we used the averaged green from the leaves as background colour.

#### 4.5.1 Technical validation

The data are mainly derived from primary data collection from the field research. All labelled plant species on botanical garden were carefully checked prior to collection. We cross validated the species identity using specific flora books (details in method section) for cross-referencing and data validation purposes. We used Taxonomic Name Resolution Service V5.0 [22] for the latest scientific name. We checked the Ocean optics spectrophotometer calibration each time before flower reflectance measurement to avoid the drift caused by electric fluctuation and external interference. Data were manually cross validated to avoid and reduce the external noise. Data were measured in dark room to avoid the external lights. We process all the raw data using same algorithm implemented in PAVO and manually cross checked with raw data and processed data. Further, we checked all data of different human colour if they are wrongly assigned to different human colour group due to colour blindness / subtleness issues of individual by plotting human colour visual model and plotted in bee and fly colour space.

#### 4.5.2 Usage notes

We used the “PAVO” package in R and the parameters associated with it in our data processing for our experimental ecology hands on practical work. Data supplied are all processed data and do not require further processing. However, we advise the users to use their own parameters, including backgrounds and photoreceptors of each insect. For example, we used the housefly *Musca dometica* photoreceptor instead of the pollinating fly *Eristalis tenax* in our R Script. *Eristalis tenax* receptor data is available in [11].

## Limitations

None.

## Ethics Statement

The current work does not involve human subjects, animal experiments, or any data collected from social media platforms.

## CRediT Author Statement

All authors discussed and designed the experiment; EH, HP, LMS, DS and MS collected the data and processed with the help of MS. Evelyn Hlawatsch, Hannah Pepe, Louis-Marvin Sander and Dietmar Schreier contributed equally on data collection and processing as part of experimental ecology research work. All authors contributed preparing the date in brief manuscript and validating the text.

## Data Availability

data_&_rcode_Shrestha_et_al. zip (Original data) (Figshare).
